# Mapping language networks and their association with verbal abilities in paediatric epilepsy using MEG and graph analysis

**DOI:** 10.1016/j.nicl.2020.102265

**Published:** 2020-04-29

**Authors:** Elaine Foley, Amanda G. Wood, Paul L. Furlong, A. Richard Walsh, Shauna Kearney, Peter Bill, Arjan Hillebrand, Stefano Seri

**Affiliations:** a School of Life and Health Sciences, Aston Brain Centre, Aston University, Birmingham, UK; b School of Psychology, Faculty of Health, Melbourne Burwood Campus, Deakin University, Geelong, Victoria, Australia; c Children's Epilepsy Surgery Service, Birmingham Women's and Children's Hospital, Birmingham, UK; d Department of Clinical Neurophysiology and MEG Center, Amsterdam Neuroscience, Amsterdam UMC, Vrije Universiteit Amsterdam, De Boelelaan 1117, Amsterdam, the Netherlands

## Abstract

Recent theoretical models of language have emphasised the importance of integration within distributed networks during language processing. This is particularly relevant to young patients with epilepsy, as the topology of the functional network and its dynamics may be altered by the disease, resulting in reorganisation of functional language networks. Thus, understanding connectivity within the language network in patients with epilepsy could provide valuable insights into healthy and pathological brain function, particularly when combined with clinical correlates. The objective of this study was to investigate interactions within the language network in a paediatric population of epilepsy patients using measures of MEG phase synchronisation and graph-theoretical analysis, and to examine their association with language abilities. Task dependent increases in connectivity were observed in fronto-temporal networks during verb generation across a group of 22 paediatric patients (9 males and 13 females; mean age 14 years). Differences in network connectivity were observed between patients with typical and atypical language representation and between patients with good and poor language abilities. In addition, node centrality in left frontal and temporal regions was significantly associated with language abilities, where patients with good language abilities had significantly higher node centrality within inferior frontal and superior temporal regions of the left hemisphere, compared to patients with poor language abilities. Our study is one of the first to apply task-based measures of MEG network synchronisation in paediatric epilepsy, and we propose that these measures of functional connectivity and node centrality could be used as tools to identify critical regions of the language network prior to epilepsy surgery.

## Highlights

• Increases in MEG connectivity were found in fronto-temporal networks during verb generation in paediatric patients with epilepsy.
• Differences in network connectivity were observed between patients with typical and atypical language representation.
• Node centrality in left frontal and temporal regions was significantly associated with language abilities.

## 1 Introduction

Normal cognitive development is characterized by an age dependent improvement across multiple cognitive abilities, including language. Childhood onset epilepsy can disrupt this systematic development, where uncontrolled seizures can have adverse effects on cognitive development, particularly if they occur during critical periods (Hermann et al., 2009). Recent evidence suggests that epileptiform activity disrupts neurophysiological synchrony within neural networks, which can alter and impair brain network development (Ibrahim et al., 2014). Resective surgery is a therapeutic option for uncontrolled paediatric epilepsy that is gaining increasing acceptance (Cross et al., 2006). However, surgery itself can lead to cognitive deficits if it entails resection of regions that support key functions, such as language and memory (de Koning et al., 2009). As a consequence, a critical step in the presurgical evaluation of paediatric patients with epilepsy is to identify language-relevant cortex and hemispheric dominance.

Traditionally, language localisation and lateralisation has been achieved using invasive methods such as the intracarotid amobarbital procedure (IAP) and electrocortical stimulation mapping (ESM). More recently non-invasive techniques such as functional magnetic resonance imaging (fMRI) and magnetoencephalography (MEG) have shown a strong potential to replace these invasive procedures in preoperative assessment (Findlay et al., 2012; Foley et al., 2019; Hirata et al., 2010; McDonald et al., 2010; Papanicolaou et al., 2004; Rodin et al., 2013). While clinical work has mostly focussed on determining hemispheric dominance or lateralisation of language function (for review see de Ribaupierre et al., 2012), recent theoretical models have emphasised the importance of integration within distributed networks in language processing (Poeppel, 2014; Price, 2012). This has resulted in a shift in approach from one that emphasises specialisation of specific regions to a network perspective that focuses on integration amongst brain regions (Sporns et al., 2005). This is particularly relevant in young patients with epilepsy as the topology of the functional network and its dynamics may be altered by the disease (Hamberger and Cole, 2011). This then has implications for the preoperative evaluation of patients with epilepsy, where understanding connectivity within the language network can provide valuable insights into healthy and pathological function, particularly when combined with clinical correlates (Chang et al., 2015; Dick and Tremblay, 2012; Duffau, 2015; Enatsu et al., 2013; Matsumoto et al., 2004).

With the advance in neuroimaging techniques and graph theoretical methods, it is now possible to explore structural and functional connectivity (FC) within the language network non-invasively. Here we will focus primarily on functional connectivity, but structural connectivity is a growing area of important research that ultimately complements functional connectivity studies of language (see Dick and Tremblay, 2012; Duffau, 2015; Friederici, 2011; Turken and Dronkers, 2011; Yamao et al., 2017). Functional connectivity analysis of resting-state fMRI, which typically defines a connection between brain regions as the presence of time-correlated fluctuations in blood oxygen levels (Biswal et al., 1995; Friston, 1994), is increasingly being used to explore cognitive networks in epilepsy (Tracy and Doucet, 2015). FC in resting-state fMRI has recently been shown to be of value in predicting strength of hemispheric lateralisation for language in adult patients with temporal lobe epilepsy (TLE) (Doucet et al., 2015). Adult patients with TLE have also shown reduced resting-state FC within the language network relative to controls (Pravatà et al., 2011; Waites et al., 2006). Furthermore, decreased resting-state FC has been associated with poorer language performance in these patients, indicating a relationship between language ability and resting-state FC (Pravatà et al., 2011).

While data on FC fMRI in paediatric patients with epilepsy is more limited, it appears to follow a similar pattern to adults, showing reduced resting-state FC within the language network (Besseling et al., 2013), and weaker resting-state intra-network integration in association with atypical language localisation (Ibrahim et al., 2015). Similarly, a task-based language fMRI study reported that children with left hemispheric focal epilepsy displayed reduced inter- and intrahemispheric functional connectivity across frontal and temporal regions compared to controls (Sepeta et al., 2015). Notably, higher FC in the left hemisphere was associated with better language ability (Sepeta et al., 2015). These FC fMRI studies have made important advances in our understanding of language networks in adults and children with epilepsy. However, FC fMRI is limited by the fact that it depends on the blood oxygenation level signal, which is an indirect measure of neural activity, and as such has relatively poor temporal resolution (in the order of seconds), due to the protracted hemodynamic response (Brookes et al., 2011). Furthermore, compared to MRI, methods based on electromagnetic measures of brain activity have the advantage of being insensitive to the distortive effects of anatomical lesions on brain microvasculature or metabolism on the developing brain (Demonet et al., 2005) and also provide a less intimidating recording environment for younger children (Pang et al., 2011).

MEG offers a non-invasive direct measurement of in vivo brain function with high temporal resolution (in the order of milliseconds), facilitating measurement of a wide range of neuronal responses. Assuming that information integration and flow are implemented by synchronised neuronal dynamics (Varela et al., 2001), then MEG adds an important dimension to decode the functioning of language processing networks. Neuronal oscillations are characterised by their spectral power and phase, and the majority of clinical MEG language studies in epilepsy have focused on the former. Studies in adults and paediatric patients with epilepsy have successfully used task-related time-varying changes in the MEG power spectrum to detect hemispheric dominance for language (Findlay et al., 2012; Foley et al., 2019; Hirata et al., 2004; Kim and Chung, 2008). While these power measures are well suited to detect “local” changes in synchronous activity, coherence or phase measures allow the investigation of interregional communication (Fries, 2005), thereby providing complementary insights into the underlying neurophysiology of the language network (Weiss and Mueller, 2012).

To date only a handful of studies have used MEG to explore language-related functional connectivity in healthy adults and children, and none in paediatric epilepsy patients. In typically developing children, broad-band connectivity (3–30 Hz) based on phase synchronisation has been found at left perisylvian sites, and hubs identified in left prefrontal regions, during a verb generation task (Youssofzadeh et al., 2017). Similarly, task-dependent increases in phase-related connectivity were observed across a broad range of frequencies (theta, alpha, and beta) during verb generation in a large group (*n* = 73) of typically developing children (4–13 years) and adolescents (14–18 years). In this group, network topology measures of strength and clustering in left prefrontal regions were significantly associated with both age and language abilities (Doesburg et al., 2015). These first studies of MEG language-related functional connectivity in typically developing children offer support for topological re-organisation of the paediatric language network during development. Notably they have shown that task-dependent FC is associated with verbal abilities. These studies form the basis for clinical applications to examine the impact of epileptiform activity on the developing language network.

In our previous work we found that beta band (13–30 Hz) spectral power reliably defines hemispheric dominance for language in paediatric epilepsy patients. However, little is known about language network synchronisation in paediatric epilepsy and how this relates to language abilities. An understanding of these network correlates could enhance clinical interpretation of language mapping during presurgical evaluation by providing a more comprehensive delineation of the patterns of neuronal synchronisation within the language network. Here we aimed to address this by investigating interactions within the language network in a paediatric population of epilepsy patients using measures of MEG phase synchronisation and graph-theoretical analysis across a broad range of frequency bands (theta, alpha, beta and gamma), and by relating these network interactions to language abilities. We hypothesised that performing a typical expressive language task would result in increased phase synchronisation between frontal and temporal language regions. Furthermore, we predicted that patients with typical and atypical language representation, defined by MEG power measures, would display significant differences in connectivity of the language network. Based on fMRI findings in epilepsy patients (Sepeta et al., 2015) and MEG findings in healthy participants (Doesburg et al., 2015), we also predicted that language abilities would be positively associated with node centrality in prefrontal regions of the dominant hemisphere.

## 2 Methods

### 2.1 Participants

Twenty-two paediatric patients with drug-resistant epilepsy under evaluation for resective surgery participated in the study. Nine male and thirteen female patients (mean age 14 yrs; SD 2.7 yrs) were included in the study (see Table 1 for a summary of patient demographics). All patients were referred to the Wellcome Laboratory for MEG studies at the Aston Brain Centre in Birmingham from Birmingham Women's and Children's Hospital NHS Foundation Trust, between 2013 and 2016, for localization of the irritative zone and eloquent cortex mapping. Inclusion criteria included being native English speakers and having demonstrated ability to perform the task during a practice session prior to the MEG recording. Focus lateralization was assessed during pre-surgical workup and was based on clinical and imaging variables, including EEG, MRI, PET and MEG where appropriate. The majority of patients had a left hemispheric focus (15/22) and the remaining 7 had right hemispheric focus (see Table 1). Just over half of the patients were right handed (13/22). It was established from seizure diary sheets that all patients were seizure-free for at least 24 h prior to MEG recording. Parents/guardians provided written informed consent for their child and all children assented to the study. The study was conducted according to the ethical principles of the declaration of Helsinki and approved by the UK NHS research ethics committee (IRAS).

**Table 1:** Summary of patient characteristics.

Pt	Age (Years)	Gender	Epilepsy Duration (Years)	Handedness	Etiology	Seizure Lateralisation	MEG Language Lateralisation	Language Abilities
1	16	F	4	R	FCD	L	L	Poor
2	16	M	6	R	TSC	R	L	Poor
3	12	F	10	R	MRI Neg	R	L	Poor
4	15	F	5	L	FCD	L	L	Good
5	11	M	9	R	FCD	R	L	Not assessed
6	15	F	7	R	TSC	L	R	Good
7	17	M	13	R	MTS	L	L	Poor
8	17	F	16	L	Stroke	L	R	Poor
9	18	F	4	L	MTS	R	B	Good
10	10	F	5	R	FCD	L	L	Poor
11	17	F	10	R	MRI Neg	L	L	Good
12	15	F	2	L	FCD	L	R	Poor
13	17	F	7	R	FCD	R	L	Good
14	11	M	3	L	FCD	L	L	Not assessed
15	14	F	13	L	MRI Neg	L	L	Good
16	16	M	5	R	FCD	L	L	Poor
17	15	F	14	L	Stroke	L	Bilat	Poor
18	15	F	10	R	MRI Neg	R	L	Good
19	12	M	11	L	MTS	L	R	Good
20	18	M	14	R	MRI Neg	L	L	Not assessed
21	14	M	12	R	Ganglioma	R	L	Good
22	8	M	6	L	FCD	L	R	Good

F female, M male, L left, R right, Bilat bilateral, TSC Tuberous Sclerosis Complex, FCD focal cortical dysplasia, MTS mesial temporal sclerosis, MRI Neg MRI negative.

### 2.2 Neuropsychological testing

Neuropsychological testing was performed on all patients as part of their routine surgical workup and included measures of language competence, IQ and handedness. These were assessed according to standardized administration of age-appropriate measures. Intellectual ability was assessed with the Wechsler Intelligence Scale for Children (WISC-IV; Wechsler, 2003) which includes measures of verbal and non-verbal abilities and provides a measure of full expressive language taskscale IQ (FSIQ). The Verbal Comprehension Index (VCI) was used as a measure of verbal ability in our analyses. Patients’ language abilities were classified as good or poor based on their overall neuropsychological language assessment and VCI scores.

### 2.3 Experimental paradigm

A ‘child-friendly’ verb generation task described in previous studies by our group was used (Fisher et al., 2008; Foley et al., 2019). In this task participants were visually presented with a series of single nouns and asked to generate an associated verb for each (e.g. ‘BALL’— ‘throw’/’catch’). To separate out component processes involved in articulation and associated muscle artefacts, participants were instructed to initially generate responses covertly and then to vocalize their response on presentation of a visual cue. The overt component of the task was included to determine that participants were performing the task correctly. The task commenced with a three-second ‘passive’ phase where participants were asked to focus on a fixation cross. Then a noun was visually presented and participants were instructed to silently generate their response (three second ‘active’ phase). This was followed by an image of Mr. Chatterbox (Copyright © 2018 THOIP - a Sanrio company) cueing participants to verbalize their response. The Mr. Chatterbox image remained on the screen for 3 s followed by an inter-stimulus interval of 500 ms before the onset of the next trial. Sixty trials were collected for this task.

### 2.4 MEG data acquisition

MEG data were recorded in a magnetically shielded room using an Elekta-Neuromag TRIUX whole-head system (Helsinki, Finland) with 204 planar dc-SQUID gradiometers and 102 magnetometers. Participants were seated in an upright position in the MEG scanner. Data were acquired with 2 KHz sampling rate, and low-pass filter of 660 Hz. One bipolar EEG channel was dedicated to recording ECG. Five coils were placed on the patient's head, three on the front and one on each mastoid for continuous monitoring of head position. To allow the translation between the MEG coordinate system and the patient's structural MRI, three head position fiducial points, at the nasion and left and right pre-auricular points, were digitized with a Polhemus Fastrack device, which was also used to digitize the surface shape of each participant's head and to digitize the location of the electromagnetic head coils with respect to that surface.

### 2.5 MEG data analysis

Artefacts were removed from the raw data with MaxFilter software (Elekta Neuromag Oy; version 2.2.10), that implements the temporal extension of signal space separation (tSSS) algorithm (Taulu and Simola, 2006). Bad channels were also identified and removed if present using MaxFilter. The cleaned data were then analysed in the Matlab R2012a environment (The MathWorks Inc., Natick, MA) using the FieldTrip toolbox (Oostenveld et al., 2011). The overt trials were initially inspected for accuracy and missed responses. Data analysis was then performed on the correct covert trials only; 5 s epochs were created based on 2.5 s pre- and 2.5 s post-stimulus. Data epochs of interest were then visually inspected for any additional artefacts caused by muscle activity and SQUID jumps and any contaminated epcohs were discarded. Trials were also inspected for interictal epileptiform discharges (IEDs) and contaminated trials were removed from further analysis. In the majority of participants (17/22) ≤10 IEDs were identified during the task and in the remaining 5 participants ≤30 IEDs were identified. On average, four noisy channels and ≤ 8 trials were removed per participant across all of the participants’ data.

### 2.6 MEG source reconstruction

High-resolution anatomical MRI scans (3D inversion recovery whole-head volume sequences were acquired with 1mm^3^ isotropic resolution) that were previously acquired within a 3-month interval at the referring centre as part of the presurgical evaluation, were used for co-registration with the MEG data. Each participant's digitized head shape was co-registered with the high-resolution anatomical MRI sequence. Co-registration was performed using in-house software based on an algorithm designed to minimize the squared Euclidean distance between the Polhemus and the MRI surfaces. The accuracy of this procedure has been shown to be within 5 mm (Adjamian et al., 2004). Realistic, single-shell brain models were constructed for each participant based on their structural MRIs (Nolte, 2003). Each patient's co-registered MRI was spatially normalized to a template MRI using the segmentation toolbox in SPM8. The automated anatomical labelling (AAL) atlas was used to define 90 cortical and subcortical regions of interest (ROIs) for further analysis (Tzourio-Mazoyer et al., 2002) after inverse transformation to the patient's co-registered MRI (Hillebrand et al., 2012).

Broadband (1–48 Hz) time series were reconstructed from each source using a time-domain linearly constrained minimum variance (LCMV) beamformer with 5% regularization (Van Veen et al., 1997). The data covariance matrix was estimated from combined active and baseline conditions for all grid points using the so-called common filter, where the data from both conditions were appended, and the covariance matrix was based on this combined data. To obtain a single time series for an ROI, we used each ROI's centroid as representative for that ROI. The centroid was defined as the voxel within the ROI that is nearest, in terms of Euclidean distance, to all other voxels in the ROI (Hillebrand et al., 2016). This atlas-based beamformer approach has been successfully used in the analysis of task-dependent MEG activity in a number of recent language studies (Doesburg et al., 2015; Youssofzadeh and Babajani-feremi, 2019; Youssofzadeh et al., 2017). Subsequent connectivity analysis was performed in four frequency bands including theta (5–8 Hz), alpha (8–12 Hz), beta (13–30 Hz) and gamma (30–48 Hz) bands.

In addition, spectral power data in the source space were computed for the beta frequency range (13–30 Hz) based on previous evidence demonstrating the key role of spectral power changes within this frequency band during language processing (Findlay et al., 2012; Fisher et al., 2008; Foley et al., 2019; Hirata et al., 2010; Pang et al., 2011). An adaptive spatial filtering beamforming technique in the frequency-domain, known as dynamic imaging of coherent sources (DICS), was used to determine sources of neuronal activity associated with the verb generation task (Gross et al., 2001). A Lateralization Index (LI) was computed based on t-values of the event related power decrease in Brodmann areas (BA) 6, 44, 45 and 22 in the left and right hemisphere (see Foley et al., 2019 for full details). These values were then used to determine hemispheric dominance for language and to classify the patients as typical or atypical representation for further analysis. We recently validated this method of determining hemispheric dominance against invasive and non-invasive measures, obtaining a high level of concordance of 89% (Foley et al., 2019).

### 2.7 Network connectivity analysis

Functional connectivity between the reconstructed signals in source space in the theta (5–8 Hz), alpha (8–12 Hz), beta (13–30 Hz) and low gamma (30–48 Hz) frequency bands were assessed using the weighted phase lag index (wPLI). The PLI measures the synchronization between time series by calculating the asymmetry of the distribution of (instantaneous) phase differences between two MEG signals, reflecting the consistency with which one signal is phase-leading or phase-lagging with respect to another signal (Stam et al., 2007). The wPLI attenuates phase synchrony occurring at zero/near-zero phase lag, which provides protection against the effect of common sources, at the cost of interpretability of the results (Vinck et al., 2011). Whole-brain connectivity patterns were assessed by computing the wPLI for all node pairs of the AAL atlas during both ‘‘active’’ and ‘‘baseline’’ periods. This produced a 90 × 90 adjacency matrix for the active and baseline conditions, for each patient averaged over epochs.

The Network Based Statistic (NBS) toolbox was then used to evaluate connectivity differences between active and baseline periods in each frequency band in order to isolate task-related connectivity effects across the group of patients (Zalesky et al., 2010). A univariate statistical threshold (t-statistic) was firstly applied to each element in the connectivity matrix to identify the size of interconnected components or clusters; a paired *t*-test was used to contrast active and baseline connectivity matrices within the group of 22 participants, testing for increases in network connectivity during task relative to the baseline period. The corrected significance of clusters (*p*<.05) was then assessed by indexing its size with the null distribution of maximal component size through permutation testing with 5000 iterations. This approach has been shown to be more sensitive than simple mass-univariate testing in connectivity studies (Zalesky et al., 2010) and has recently been applied in a MEG language study on typically developing children (Doesburg et al., 2015).

A similar approach was used to compare patterns of network connectivity between patients with typical and atypical language representation. Baseline connectivity matrices were subtracted from active matrices, and the NBS was used to statistically compare the resulting task-related connectivity matrices between the two groups using an independent *t*-test. Patterns of network connectivity between patients with good and poor language abilities were also assessed using the NBS. Again, baseline connectivity matrices were subtracted from active matrices, and the NBS was used to statistically compare the resulting task-related connectivity matrices between the two groups while controlling for hemispheric lateralization and age (ANCOVA).

The Brain Connectivity toolbox (Rubinov and Sporns, 2010) was used to compute measures of centrality for regions of the language network identified during NBS analysis. Betweenness centrality (BC) was computed from the 90 × 90 wPLI adjacency matrices for each frequency band and results were visualised using BrainNet Viewer (Xia et al., 2013). Betweenness centrality calculates the shortest (weighted) path between every pair of nodes in a connected graph and is defined as the tendency for a node to occupy positions along these shortest paths (Freeman, 1977; Bonacich, 2007). It provides a good representation of influential nodes in a network (Hagmann et al., 2008; Power et al., 2010). Spearman's correlation was used to investigate the relationship between betweenness centrality in the beta band and language abilities (VCI), and between beta oscillatory power and language abilities.

## 3 Results

### 3.1 Language network connectivity during verb generation

**Fig. 1:**
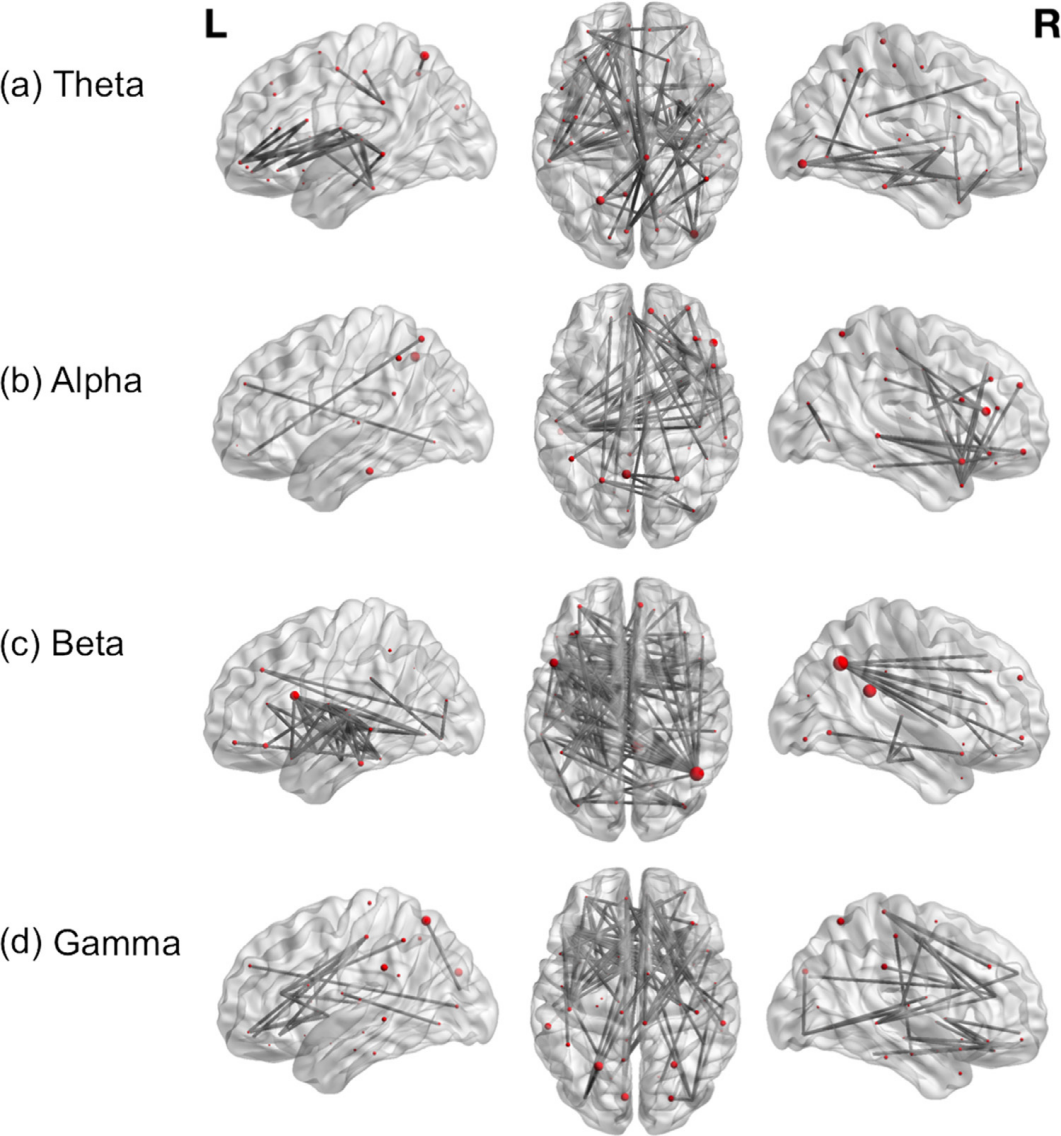
Group-level (*n* = 22) functional connectivity in (a) Theta (b) Alpha (c) Beta (d) Gamma bands during verb generation, displayed on a template brain (t(21)=3; *p*<.05). Dark grey lines indicate significant connections between brain regions, which are depicted as spheres. The size of each sphere denotes task-dependent increases in centrality. *L*= left; *R* = right.

All of the patients performed the verb generation task successfully, with at least 95% of the trials correctly completed. This was judged on the basis of their overt responses to the stimuli. Across our group of 22 patients we found significant increases in phase synchronisation between regions of the language network during verb generation (t(21) = 3; *p*<.05; see Fig. 1). In theta and beta bands in particular, significant connectivity was observed in the left hemisphere between typical regions of the language network, including Broca's area in the inferior frontal gyrus (IFG) incorporating pars opercularis and triangularis; Wernicke's area incorporating inferior and superior temporal gyri (STG) and the supramarginal gyrus; the angular gyrus; and inferior temporal regions or the so-called ‘basal’ temporal language area. In the right hemisphere, there was predominantly long-range connectivity between the angular gyrus and inferior and middle frontal regions. Notably there was significant interhemispheric connectivity between frontal and temporal regions (see Fig. 1). Significant connectivity between frontal and parietal regions was more prominent in alpha and gamma frequency bands.

**Fig. 2:**
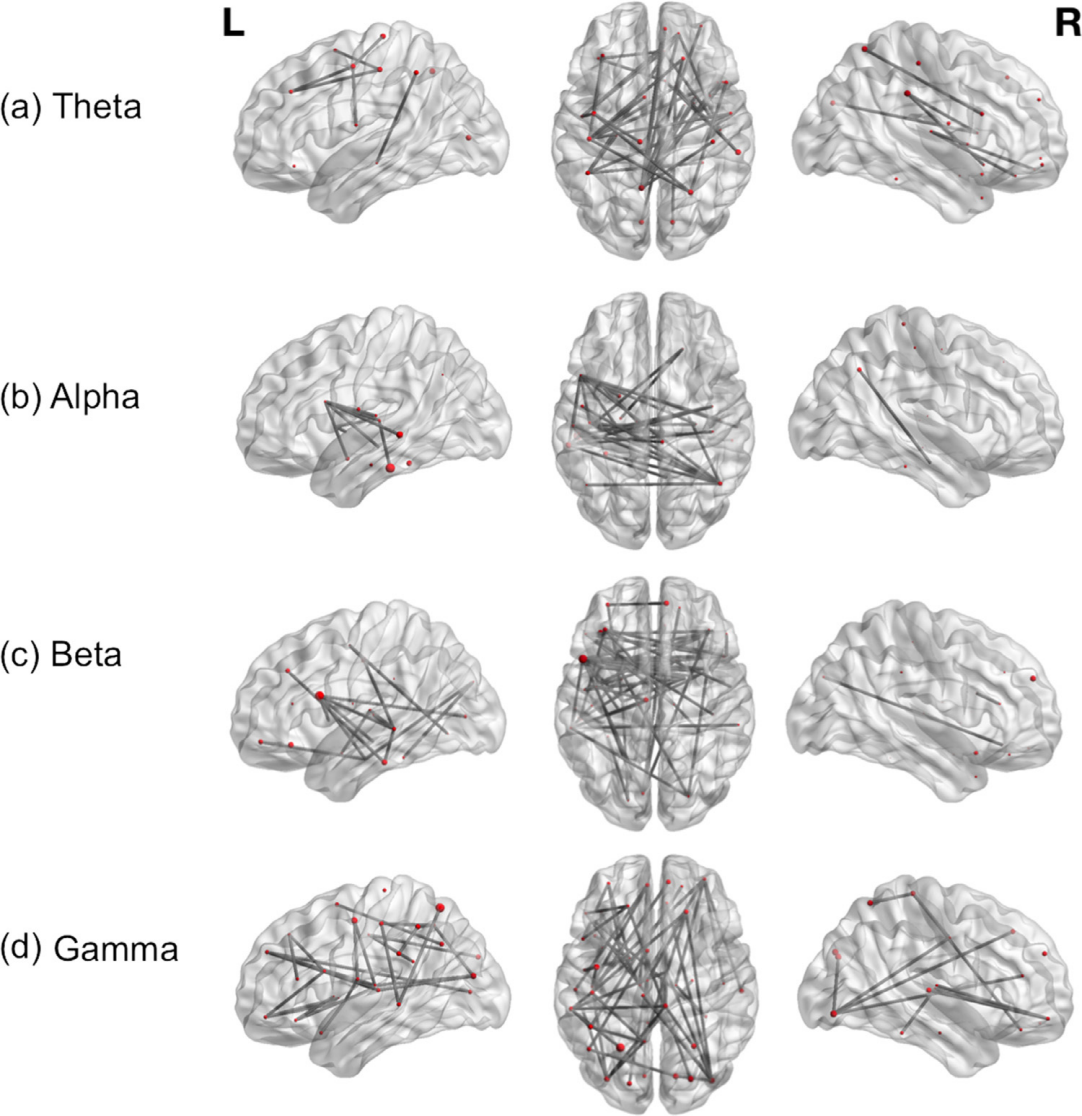
Contrast of patients with typical versus atypical language representation. Functional connectivity in (a) Theta (b) Alpha (c) Beta and(d) Gamma bands, during verb generation, is displayed on a template brain (t(21)=3; *p*<.05), dark grey lines indicate significant connections between brain regions, which are depicted as spheres. The size of each sphere denotes task-dependent increases in centrality. *L*= left; *R* = right.

**Fig. 3:**
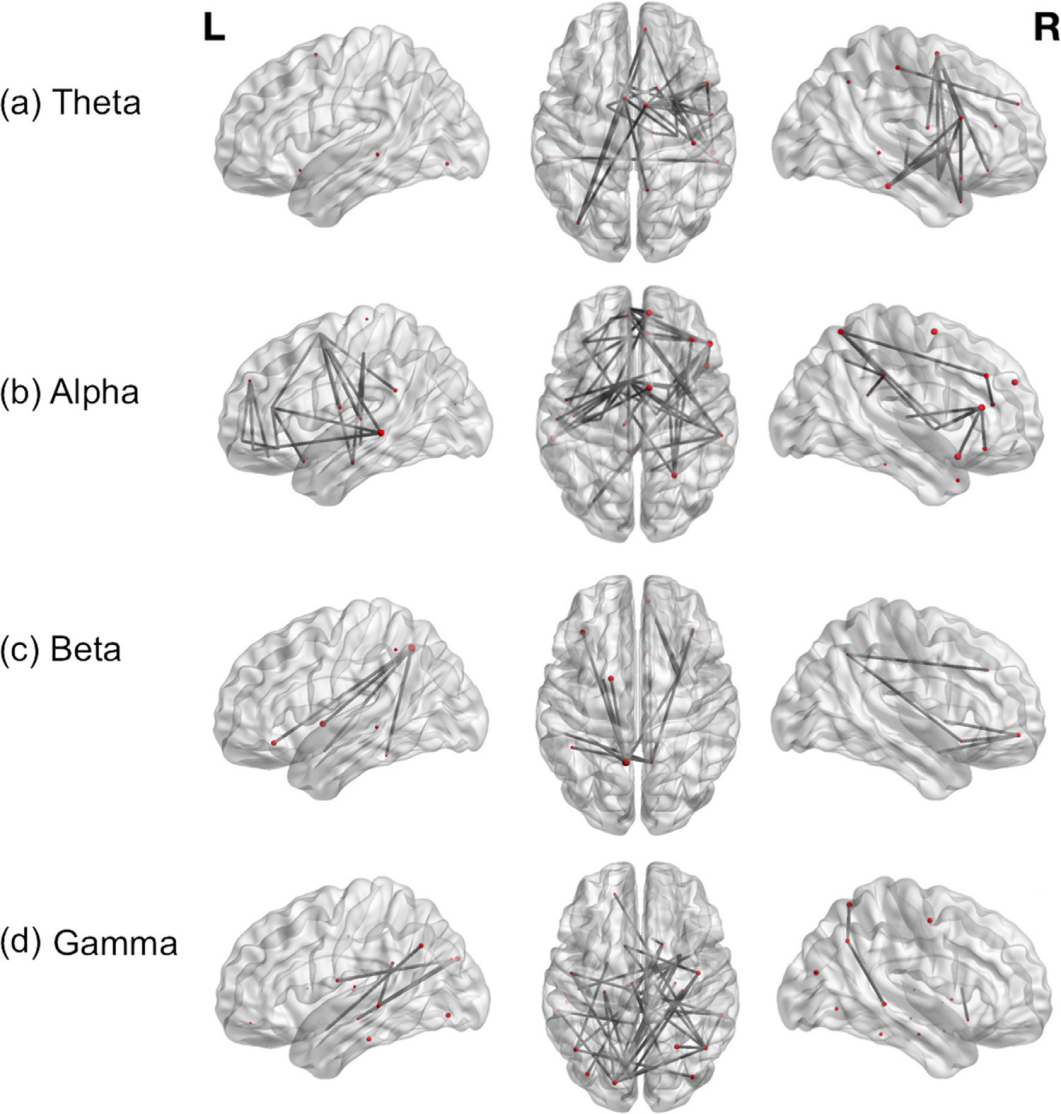
Contrast of patients with atypical versus typical language representation. Functional connectivity in (a) Theta (b) Alpha (c) Beta and (d) Gamma bands, during verb generation, is displayed on a template brain (t(21)=3; *p*<.05), dark grey lines indicate significant connections between brain regions, which are depicted as spheres. The size of each sphere denotes task-dependent increases in centrality. *L*= left; *R* = right.

Approximately 30% of patients were classified as having atypical language representation i.e. they had bilateral or right hemispheric dominance based on their MEG lateralisation index (see Table 1), which could have affected the overall group network connectivity measures. We therefore assessed differences in task-related connectivity between patients with typical and atypical language representation (t(21)=3, *p*<.05). Patients with typical language representation (*n* = 15) showed similar network connectivity during verb generation to the whole group results across the four different frequency bands, with significant intra-hemispheric connections in the left hemisphere between fronto-temporal regions and fronto-parietal regions, relatively few right intra-hemispheric connections and considerable inter-hemispheric connectivity particularly between the frontal and temporal lobes (see Fig. 2). Conversely, patients with atypical language lateralisation (*n* = 7) had fewer left intra-hemispheric connections but significant right intra-hemispheric connections between fronto-temporal regions and mainly fronto-temporal inter-hemispheric connectivity (see Fig. 3).

### 3.2 Network connectivity and node centrality association with verbal abilities

**Fig. 4:**
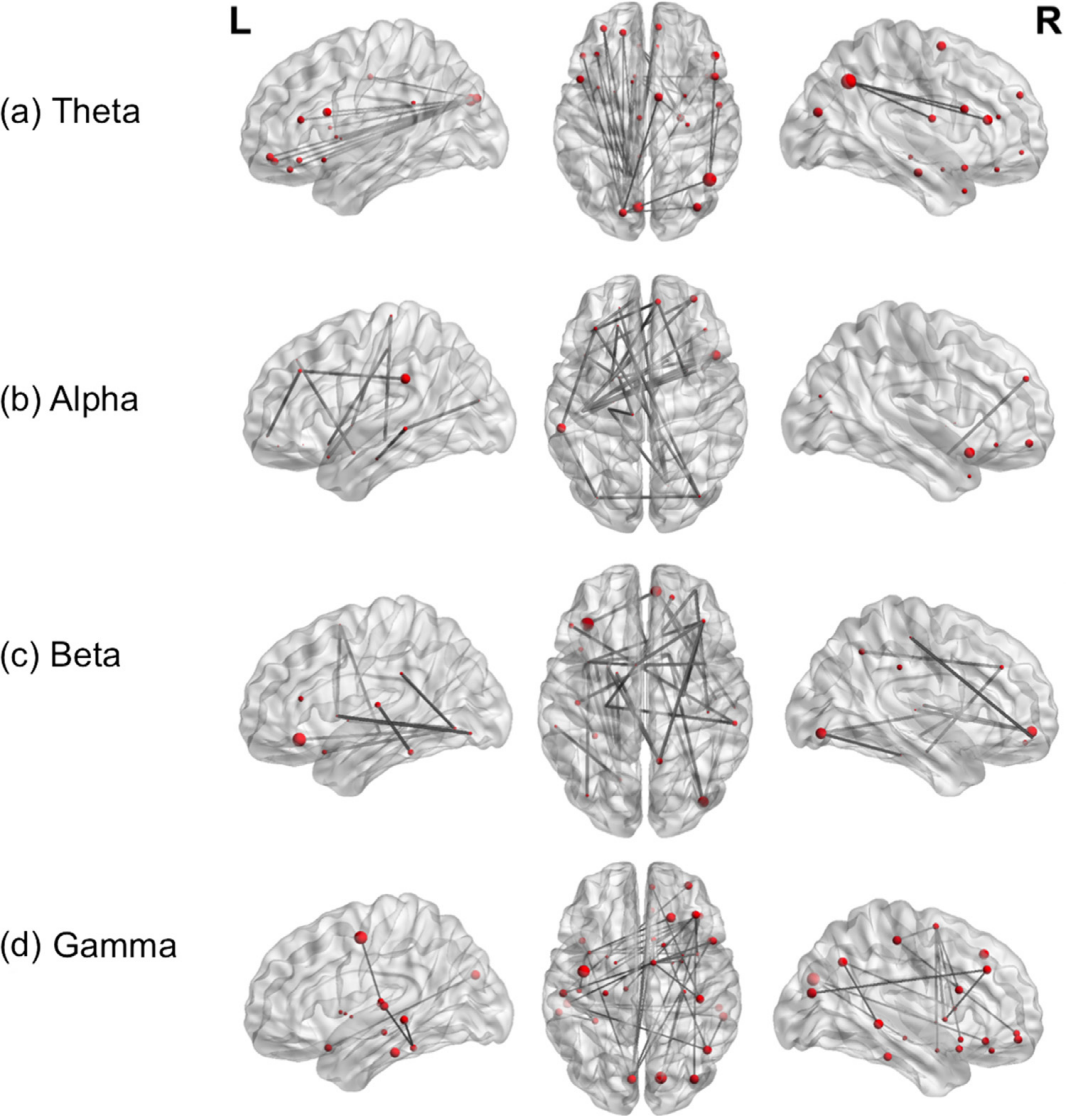
Contrast of patients with good versus poor language abilities with hemispheric lateralisation and age included as covariates. Functional connectivity in (a) Theta (b) Alpha (c) Beta and (d) Gamma bands, during verb generation, is displayed on a template brain (t(18)=3; *p*<.05), dark grey lines indicate significant connections between brain regions, which are depicted as spheres. The size of each sphere denotes task-dependent increases in centrality. *L*= left; *R* = right.

Neuropsychological testing was performed in all of the patients prior to MEG recording, but VCI measures were not obtained in 3 patients due to failure to complete the tests. We therefore included only those 19 patients with VCI scores in the following analyses. Patients’ language abilities were classified as good or poor by a clinical neuropsychologist based on their overall neuropsychological language assessment and VCI scores. Patients were classified as having poor language abilities if their scores fell significantly below the normative mean. In this cohort 10 patients were identified as having good language abilities and 9 patients had poor language abilities. Significant interhemispheric connectivity was observed for patients with good relative to those with poor language abilities across the four frequency bands, particularly between temporal and frontal regions, while controlling for age and language lateralization (see Fig. 4). Common nodes showing significant increased connectivity and centrality were identified in left inferior frontal regions in theta, alpha and beta frequency bands and in left temporal regions in alpha, beta and gamma bands. These nodes were therefore used in subsequent analysis of betweeness centrality in the beta band.

In order to compare the relationship between language scores and graph topological measures relative to power measures we focused our subsequent analysis on the beta band only. Language abilities (VCI) were significantly positively correlated with betweenness centrality of the nodes in the left inferior frontal gyrus (r(17)=0.41, *p*=.04) and left superior temporal gyrus (r(17)=0.52, *p*=.02) in the beta band. There was no significant correlation between VCI and measures of beta oscillatory power in these regions. There were no significant differences in MEG beta oscillatory power between patients with good and poor language abilities. There was no significant difference in duration of epilepsy between the two groups either, and epilepsy duration did not correlate with node centrality in any of the ROIs, nor was it related to power measures or LI. Furthermore, rate of IEDs did not correlate with node centrality in any of the ROIs and there was no relationship between IED rate and VCI scores. Absence of significance however has to be interpreted with caution; given the relatively small number of participants we cannot exclude the possibility of a type II error.

## 4 Discussion

We have demonstrated significant increases in broad-band connectivity between regions of the language network during expressive language processing in a group of paediatric epilepsy patients. We used a whole-brain connectivity approach rather than focusing on predefined regions of interest in order to incorporate and explore the broad network of regions involved in language processing. Across the group of 22 patients, significant task-based connectivity was observed in the left hemisphere between important regions of the language network, including Broca's area incorporating pars opercularis and triangularis; Wernicke's area incorporating inferior and superior temporal gyri (STG) and the supramarginal gyrus; the angular gyrus; and inferior temporal regions commonly referred to as the ‘basal’ temporal language area. All of these regions are commonly associated with language processing and have been identified in numerous MEG language studies in healthy participants (Doesburg et al., 2015; Schoffelen et al., 2017; Youssofzadeh and Babajani-Feremi, 2019; Youssofzadeh et al., 2017) and patients (Foley et al., 2019; Hirata et al., 2010) using various language tasks. These regions also form part of a clinical language model that has recently been proposed to highlight critical regions for fMRI language mapping studies (see Benjamin et al., 2017). These authors advised that resection of these key regions may lead to language deficits, irrespective of their hypothesised function. Our MEG data support this model and provide additional information on the potential mechanisms of communication within this network via synchronisation of a range of rhythms.

MEG measurements are dominated by neural oscillations which occur at multiple temporal scales, ranging from 1 Hz to ~200 Hz. Phase synchronisation of neural oscillations is believed to be the underlying mechanism facilitating binding and information transfer between distributed brain regions involved in cortical processing (Buzsáki and Draguhn, 2004; Fries, 2005, 2015; Roopun et al., 2008). MEG is particularly well-suited to the investigation of phase synchrony as it permits recordings on a temporal scale in the order of milliseconds (Hamalainen et al., 1993). Previous MEG studies in typically developing children have reported increased task-dependent phase synchronisation between fronto-temporal regions in a broad frequency range (3–30 Hz) during language processing (Doesburg et al., 2015; Youssofzadeh et al., 2017). Our findings are in line with these studies and demonstrate how MEG connectivity measures can be applied to a clinical paediatric epilepsy population.

There is likely a complex interplay between different frequency bands, and multiple rhythms appear to be involved in language processing (Schoffelen et al., 2017; Weiss and Mueller, 2012). It is believed that distant brain areas are more likely to synchronise in beta frequencies, as such beta frequency oscillations have been linked to various aspects of language processing, including top-down mechanisms (Weiss and Mueller, 2012), possibly facilitated by propagation in beta rhythmic activity from frontal to temporal regions (Schoffelen et al., 2017). Our findings revealed relatively similar patterns of connectivity in beta and theta bands across the group, particularly between left fronto-temporal regions (see Fig. 1). Low-frequency oscillations such as those in the theta band are also believed to be particularly relevant for long-range interactions (von Stein and Sarnthein, 2000). Theta oscillations have been implicated in expressive language processing (Hermes et al., 2014; Piai et al., 2014; Doesburg et al., 2015) and theta-band synchronisation has been reported during word reading (Bedo et al., 2014). Notably, Doesburg et al. (2015) found that increased connectivity was most pronounced in the theta frequency range during verb generation in typically developing individuals. Modulation of gamma activity by theta phase has also been reported during a verb generation task (Doesburg et al., 2012).

According to the traditional view, language processing primarily involves the left hemisphere in healthy adult participants (typical representation) with variation associated with left-handedness (Knecht et al., 2000). However, there is a higher incidence of atypical representation (bilateral or right lateralised) in epilepsy patients and in children (Berl et al., 2014). While atypical language patterns are less frequently present they are more variable, and consequently more difficult to interpret, particularly in paediatric patients. Recent work has demonstrated many different patterns of language representation and crossed dominance, supporting the notion that language function should be assessed on a regional or network level as opposed to a hemispheric one (Balter et al., 2016; Berl et al., 2014). In line with this, we conducted connectivity analysis to explore variation in the networks underlying typical and atypical language representation.

Hemispheric dominance for language was determined using measures of beta oscillatory power, which were validated against clinical findings in a previous study (Foley et al., 2019). Approximately 30% of patients in the current study were classified as having atypical language representation. Long-range intra-hemispheric phase synchronisation in alpha and beta bands was observed between frontal and temporal regions predominantly in the left hemisphere, and between frontal, temporal and parietal regions in theta and gamma bands for the group of patients (*n* = 15) who were classified as left hemisphere dominant. Significant intra-hemispheric phase synchronisation between frontal, parietal and temporal regions was identified in the right hemisphere for the group (*n* = 7) who were classified as having atypical language representation i.e. right hemisphere dominant or bilateral. A recent study in healthy adults has shown that interactions from frontal to temporal regions were subserved by beta synchronisation at ~27 Hz during a word-reading task (Schoffelen et al., 2017), whereas interactions in the opposite direction were related to alpha synchronisation (~12 Hz). While our analyses do not provide information on the direction of information flow between these regions, it is possible that the alpha and beta phase synchronisations described here facilitate information flow from frontal to temporal regions in both hemispheres during expressive language processing, which may represent mechanisms of top down processing (Weiss and Mueller, 2012).

Notably, there was significant interhemispheric functional connectivity in both groups of patients. This may reflect the paediatric sample, as connectivity within the language network changes from predominantly inter- to intrahemispheric connections during development, with an increase in long-range intrahemispheric connections between frontal and temporal regions within the left hemisphere during normal development from childhood to adulthood (Friederici et al., 2011; Xiao et al., 2016). Given that the age range of our sample is relatively broad (8–18 years) it could capture this developmental trajectory, however future research with longitudinal imaging acquisition would better support this interpretation. More broadly, these findings are consistent with growing evidence in support of the essential role of the right hemisphere in language processing in general (Gajardo-Vidal et al., 2018). Evidence demonstrating the role of bilateral anterior temporal lobes in language processing is accumulating, as is evidence for the requirement of the right inferior frontal cortex for language comprehension (Gajardo-Vidal et al., 2018). This highlights the importance of considering both intra- and interhemispheric connectivity to better characterise the language networks in paediatric patients.

In addition, patients with good language abilities showed significant interhemispheric connectivity in all four frequency bands, relative to patients with poor language abilities, regardless of hemispheric laterality. This suggests that connectivity within the language network plays an important role and may be directly associated with language abilities. These patients were dichotomized as having either good or poor language abilities to reflect the reality of clinical decision-making, with the aim to make the insights gained by this approach more readily translatable into information that can support the interpretation of task-based connectivity in a clinical setting.

Recent studies in MEG and fMRI have reported associations between language abilities and measures of network centrality in typically developing children (Doesburg et al., 2015) and patients with epilepsy (Sepeta et al., 2015). Consistent with this, we found that patients’ language abilities were associated with increased node centrality in left inferior frontal and superior temporal regions in the beta band, where patients with good language abilities had significantly greater node centrality in left IFG and STG compared to patients with poor language abilities. Centrality of a node indicates its structural or functional importance within a network, where highly central nodes may serve as centres of information integration (Power et al., 2010). Notably, we did not find an association between language abilities and MEG power measures in these regions.

We focused our comparative analysis of node centrality and power specifically in the beta band (13–30 Hz) as beta oscillatory power has been shown to be a clinically relevant measure of hemispheric dominance in a large cohort of adult epilepsy patients (Hirata et al., 2010) and in paediatric epilepsy patients (Foley et al., 2019). Previous studies have highlighted the value of MEG for language mapping during presurgical evaluation using various analysis methods that were generally based on spectral power measures (Foley et al., 2019; Hirata et al., 2010; McDonald et al., 2010; Papanicolaou et al., 2004). Our current findings highlight the complementary nature of functional connectivity, network topology, and power measures and demonstrate that measures of network centrality may provide a more sensitive means of detecting neurophysiological associations with cognitive abilities.

Previous MEG studies have reported increased centrality in left inferior frontal regions during development, where adolescents displayed increased centrality in left IFG compared to children (Youssofzadeh et al., 2017). Similarly, Doesburg et al. (2015) found that age and language abilities were associated with connectivity strength and clustering in left prefrontal regions as well as left supramarginal and angular gyri in typically developing children and adolescents. An fMRI language study of paediatric epilepsy patients reported a correlation between language performance and increased connectivity between IFG and Wernicke's area (Sepeta et al., 2015). Taken together, these findings suggest that connectivity and centrality of left frontal and temporal language areas has a developmental pattern and is related to cognitive ability. This is of particular importance when assessing paediatric patients, where measures of connectivity and node centrality could be used as additional tools to identify critical regions of the language network prior to surgery. This is in line with a recent study in a group of healthy adult participants that found good spatial concordance between fMRI and MEG connectivity measures of expressive and receptive language processing, and who highlighted that an important application of MEG functional network analysis is in determining language lateralisation in neurosurgical candidates (Youssofzadeh and Babajani-feremi, 2019).

A limitation of the current study is the heterogeneous sample, which included patients with left and right hemispheric focal epilepsy with mixed language dominance. In order to explore network differences in patients with typical and atypical language dominance we chose to include both types of patients in this study. Although the number of patients in each group was relatively small, our results begin to shed light on the neurophysiological underpinnings of language network connectivity in patients with typical and atypical language representation. We did not include typically developing controls in this study and therefore our findings may not be epilepsy-specific. It must also be noted that IEDs have been shown to affect connectivity measures in resting state networks of epilepsy patients (Shamshiri et al., 2017). We tried to account for any potential effects of IEDs on our connectivity analyses by removing any trials containing IEDs from our analysis. In addition, the majority of patients included in this study had relatively low rates of IEDs. We also did not find a relationship between IED rate and verbal abilities or betweenness centrality. Future studies with larger numbers of participants could further elucidate network differences between typical and atypical language representation, both in healthy individuals and in patients.

It must also be acknowledged that MEG based characterisation of language network connectivity is in fact complicated by the rich information content of electrophysiological signals. In this study we explored task-related connectivity in multiple frequency bands separately. However recent studies have proposed more integrated analysis strategies based on, for example multi-layer networks, to provide a single framework in which to combine within-band and cross frequency interactions, and thereby represents the brain as a single multi-dimensional network (Brookes et al., 2016). This could be an important avenue of future language network research in epilepsy that could build upon our current findings. Furthermore, the effects of surgery on these networks would also be a key area of future work, namely to explore network changes and reorganisation following surgery and associations with language abilities. Based on our findings we suggest that measures of functional connectivity and node centrality could be used as tools to identify critical regions of the language network prior to epilepsy surgery, where post-operative outcome data would ideally be necessary to provide supporting evidence for this hypothesis.

In conclusion, we have demonstrated that task-based MEG connectivity and measures of network topology can provide insights into the underlying function and integration within the complex language network in paediatric epilepsy. Furthermore, we have shown significant interhemispheric connectivity in patients with good language abilities relative to those with poor, and that greater node centrality in left IFG and STG is related to better language abilities, which may be particularly relevant when assessing paediatric patients prior to surgery. Our study is one of the first to apply task-based measures of network synchronisation in paediatric epilepsy, and demonstrates the potential added value of a MEG-based network approach during the presurgical evaluation process.

## CRediT authorship contribution statement

**Elaine Foley:** Conceptualization, Methodology, Visualization, Writing - original draft. **Amanda G. Wood:** Supervision, Writing - review & editing. **Paul L. Furlong:** Resources, Writing - review & editing. **A. Richard Walsh:** Validation, Writing - review & editing. **Shauna Kearney:** Resources, Writing - review & editing. **Peter Bill:** Resources, Data curation, Validation, Writing - review & editing. **Arjan Hillebrand:** Software, Methodology, Writing - review & editing. **Stefano Seri:** Supervision, Resources, Validation, Writing - review & editing.

## Declaration of competing interest

None.
